# Community-Based Mental Health Intervention for Underprivileged Women in Rural India: An Experiential Report

**DOI:** 10.1155/2011/621426

**Published:** 2011-08-09

**Authors:** Kiran Rao, Prameela Vanguri, Smita Premchander

**Affiliations:** Sampark, No. 80 Ground Floor, 2nd Main Road, 1st Block, Koramangala, Bangalore 560 034, Karnataka, India

## Abstract

*Objective*. To share experiences from a project that integrates a mental health intervention within a developmental framework of microcredit activity for economically underprivileged women in rural India. *Method*. The mental health intervention had two components: group counseling and stress management. The former comprised of ventilation and reassurance and the latter strengthening of coping skills and a relaxation technique. Focus group discussions were used to understand women's perception of how microcredit economic activity and the mental health intervention had affected their lives. *Results*. Women in the mental health intervention group reported reduction in psychological distress and bodily aches and pains. Majority (86%) reported that the quality of their sleep had improved with regular practice of relaxation and that sharing their problems in the group had helped them to unburden. The social support extended by the members to each other, made them feel that they were not alone and could face any life situation. *Conclusion*. The study provided qualitative evidence that adding the mental health intervention to the ongoing economic activity had made a positive difference in the lives of the women. Addressing mental health concerns along with livelihood initiatives can help to enhance both economic and social capital in rural poor women.

## 1. Introduction

India accounts for 15 percent of the global population and one-third of the world's poor. In 2009, more than 200 million households were described as living in absolute poverty in India [1]. Emotional distress and depression, manifested mainly through somatic symptoms, are strongly associated with poverty, especially in developing countries [2–6]. Community and clinic-based studies in India have shown that women are twice as likely to be affected by depression as men [7]. There are a number of potential factors for this increased gender vulnerability: poverty, social class, marital and childbearing roles, lack of education, and social oppression [8, 9]. However, most of this morbidity is unrecognized and untreated [10]. Women tend not to seek help because of stigma, poverty, lack of awareness, and lack of access to care. Even when women are able to access care and are prescribed antidepressant medication, they drop out of treatment due to side effects of medication and the costs of continuing treatment [11]. Thus, psychological distress poses a major economic burden on poor women and adversely impacts their livelihoods. Counseling, coping techniques, and breathing exercises are found to be useful in the management of depression in the community [12, 13]. However, individual interventions are resource intensive, especially in developing countries, where trained mental health professionals are few in number. The National Mental Health Program (NMHP) in India, concerned with the delivery of basic mental health services, has mainly focused on severe mental disorders [14]. The treatment of depression, especially in rural communities, has largely been neglected. 

This paper is an experiential report of an ongoing project in which a mental health intervention has been introduced within a well grounded economic support program. The project was designed and implemented by Sampark (India), a registered community-based, nonprofit, nongovernmental organization (http://www.sampark.org/). Sampark has been working for the past 20 years in various parts of India, in the area of poverty alleviation, especially with women from vulnerable and disadvantaged sections [15]. Sampark raises funds for project activities from both national and international donor organizations.

## 2. Background to the Project

The present project was conducted in Koppal, a district located in the State of Karnataka in the Southern part of India. Koppal is a semiarid, drought-prone region with a population of about 1 million people spread over 488 villages. Livelihood is mainly dependent on rain-fed agriculture. The composite human and gender development index is low, and many households live below the poverty line [16].

### 2.1. Poverty Alleviation through Microcredit Activities Using Self-Help Groups

Sampark staff helps underprivileged, rural poor women to form savings and credit self-help groups (SHGs) and trains them in the management of SHGs (numeracy skills, money management, and book keeping), vocational skills, and enterprise development. SHGs are small groups (typically 15–20) of poor persons who voluntarily come together to form a group based on the principles of thrift/savings, credit, and self-help. Members of the SHG agree to save small amounts of money (In India, about half to one dollar equivalent) regularly (usually weekly, sometimes daily) and contribute to a common fund. The group uses the common fund and other funds, such as loans from banks, to give small loans (known as microcredit) to needy members. SHGs are self-governing organizations, and members determine their own rates of interest for savings and loan. Members are jointly liable for each other's loans and use peer pressure to ensure proper end use of credit as well as timely repayment of loans. Microcredit policies are based on the premise that people are poor, not due to lack of skills, but due to the absence of an enabling environment. Microcredit helps poor people to start microenterprises and raise their income levels and standard of living. Globally, the majority of the SHGs comprise women, and India is no exception. The government of India, through a national bank (NABARD), has linked the SHG movement with formal financial institutions and recognizes the facilitator role played by NGOs [17]. To date, Sampark has established 230 SHGs involving about 3500 women in 35 villages of Koppal district.

### 2.2. Poverty and Mental Health

While focusing on income generation activities and poverty reduction, the staff of Sampark observed that a number of women reported somatic complaints such as body aches and pains, disturbed sleep, tiredness, and a variety of gynecological symptoms. The women were under chronic stress due to poverty and social exclusion and felt helpless and hopeless about their future. Although distressed by their health status, they did not seek formal medical or psychological help for these complaints due to stigma and lack of access to formal services. To address this concern, Sampark sought to pilot test a mental health intervention with the ongoing SHG activity in 12 SHGs in two villages (Kolur and Gunnalli) in Koppal.

### 2.3. Mental Health Intervention to Address Psychological Distress

Mental health professionals trained the Sampark staff at Koppal in basic techniques of counseling and stress management [18]. Details of the training are provided in Appendix A. Prior to introducing the mental health intervention, a health screening camp was held at the village level for SHG members and their families in order to identify and treat/refer women with a primary medical illness or a serious psychiatric illness. The typical *medical* problems noted among women attending the camp were severe anemia, primarily due to worm infestations, and reproductive health problems, similar to other community surveys in India [3]. 

Following the health camp, in each SHG of the identified villages, the mental health intervention was introduced as a group session on a fortnightly basis and conducted after the savings and credit discussion of that week had been completed. Participation in the counseling session was kept voluntary. The first ten sessions were facilitated by the Sampark staff. During these 10 sessions, the group identified two members who could function as group facilitators or moderators. Group facilitators were then trained by the Sampark staff and provided ongoing supervision and booster training. Subsequent sessions were conducted by the group facilitators, thus ensuring that the intervention was sustained. Each session of the mental health intervention comprised of a group song (specifically written and set to tune by the local women), followed by sharing and ventilation by individuals in the group and reassurance, problem- and emotion-focused coping suggestions provided by group members. The session ended with the practice of a breathing-based relaxation exercise. The song was mainly to facilitate the transition from economic matters to personal concerns, with the verses emphasizing that life was a cycle of joy and sorrow and sharing and problem-solving in the group could help one face it better. Details of the mental health intervention are provided in Appendix B.

## 3. Material and Methods

### 3.1. The Present Study

A qualitative research approach was used to address the following objective: to understand the women's perception of integrating a mental health intervention with their ongoing SHG activity. 

### 3.2. Sample

The sample comprised two groups. Group I comprised women from 2 villages (Kollur and Gunnalli) who had been involved in savings and credit SHG activity for about 4 years and had been practicing the mental health intervention for about a year. There were 12 SHGs in these two villages comprising in all 180 members. Group II comprised women from 2 villages (Madanur and Mornal) who had been involved in SHG activity for a period of about 4 years, similar to Group I but had not yet been introduced to the mental health intervention. There were 10 SHGs in these two villages comprising in all 160 members. Group II formed the comparison/control group. Sampark staff visited all of the above-identified SHGs and verbally explained the purpose of the study and the time involved and requested members to indicate their willingness to participate by raising their hands. Majority indicated their willingness to participate, with the exception of one or two women in each group. The main reason for nonparticipation was severe time constraints. From among the women who had indicated their consent, two women were randomly selected to represent the group by picking lots. They were given further information regarding the date, time and venue of the study. The study was conducted using the focus group discussion (FGD) method detailed below.

### 3.3. Tools

1. Focus group discussion is a qualitative research technique that generates data through interaction among the participants. In responding to each other, participants reveal more of their own frame of reference on the subject of study and are less influenced by interaction with the researcher [19]. FGD was chosen as participants lacked literacy skills for any kind of quantitative assessment.
2. The specific issues raised in the FGD were
  - women's perception of how savings and credit activity in the SHG had affected their lives,
  - women's perception of the usefulness of the mental health intervention.

### 3.4. Method

Separate FGDs were conducted for Group I and Group II. The FGDs were held in the premises of a government school building in a village close to where the SHGs were located. Both FGDs were conducted on a Sunday in the last two weeks of October 2009. Sunday being a school holiday, there was adequate privacy for the discussion to take place. Participants sat cross-legged on the floor in a circle. The FGDs took place in the vernacular language (Kannada) and were led by the second author (P. Vanguri), while the group interaction was noted down verbatim by the first author (K. Rao) as well as by two of the Sampark staff. Each of the FGDs lasted for about two hours. Initial few minutes were spent in allowing members to interact with each other and settle down. The second author (P. Vanguri) then explained the purpose of the FGD and how the FGD functioned. All participants were encouraged to express their views and not merely agree with one another. Consensus and agreement among group members concerning a particular opinion was often taken with a show of hands. After the FGDs were completed, the responses and themes that had emerged in the discussion were identified by the first and second author, along with the Sampark staff. The main findings are presented in the results section.

### 3.5. Ethical Issues

Participation in the FGD was voluntary. Informed consent was obtained verbally from each participant. They were clearly informed that they were free to leave the group discussion at any time. All participants, being daily wage earners, were economically compensated for their time (loss of wages incurred as a result of being unable to go to work for one shift) and travel costs, if any, and were provided tea and snacks during the discussion. The project was implemented in accordance with the ethical guidelines for human subjects in the Declaration of Helsinki (1964). 

## 4. Results

In Group I, 21 of the 24 participants who had volunteered to participate attended the FGD. There were 2 participants each from 9 SHGs and one participant each from three SHGs. In Group II, 16 of the 20 participants who had volunteered attended the FGD. There were 2 participants each from 7 SHGs and one participant each from 2 SHGs. Paucity of time (*N* = 5), child not well (*N* = 1), and religious function in the family (*N* = 1) were the reasons given by the participants who did not come for the FGD. Women in both groups were in the age range of 20 to 65 years. 

### 4.1. Women's Perception of the Impact of SHG Activity

Women in both groups reported that at the start of the SHG activity, they were able to save only Rs 10/- (approx. $0.25) per week, but four years hence, they were now able to save Rs. 20 to Rs. 50 (approx. $0.50 to $1) per week. The women indicated that they earned more interest for the money that they saved and were able to easily access loans at lower rates of interest. As a result, they were no longer indebted to the local money lender who charged a much higher rate of interest on loans. Women in both groups generally indicated that initial loans from the SHG had been taken to meet household emergencies such as illness in the family, school fees of a child, or house repairs. However, good credit discipline through regular repayment of the loans to the SHG had enabled them to get larger loans from the bank. While most of the women still worked as daily wage earners during the agricultural season, the majority of the women in Group I (18/21, 85%) and 40% (6/16) of the women in Group II had started their own small business, such as operating a small grocery store, selling hand-pounded spices or food products, dairy farming, or rearing a few goats/sheep for sale. The remaining women stated that they had invested in agriculture as they had small land holdings.

About one-fourth of the women in both the groups (5/21 in Group I and 4/16 in Group II) mentioned that they had seen a bank prior to joining the SHG, while the remaining stated that they did not even know what a bank was till they became a member of the SHG. Now, each member operated a bank account and was able to interact with the staff at the bank. 

All the women in both the groups reported that joining the SHG and participating in savings and credit activities had increased their income generating capacity. This had led to greater economic security and stability. With seeming uniformity of opinion, the women in both the groups indicated that their ability to contribute substantially to the family income and raise money to meet important or unexpected expenses had earned them the respect of their spouse and other family members (especially in-laws). They were now consulted and involved in decision-making processes within the family such as the purchase of goods (usually a television) or the education/marriage of their children.

### 4.2. Women's Perception of the Mental Health Intervention

Women in Group I stated that as a result of participating in the mental health intervention, their general health had improved. The majority of the participants (*N* = 18/21, 86%) reported that the quality of their sleep had improved with regular practice of the relaxation exercise and they were able to carry out their routine work without feeling tired or suffering from bodily aches and pains. Three women stated that they were not able to practice the relaxation at home due to time and space constraints and did so only in the fortnightly group session and, hence, may not have benefitted like the others. All the women indicated that after the psycho-educational session, they realized that many of their somatic complaints were a manifestation of their mental worries and distress. Interpersonal problems, mostly within the family (spouse, parents, in-laws, children, and other relatives), not having a child or not having a male child, poverty, and social barriers of exclusion were the most common sources of stress. Sharing their problems with each other in their SHG had helped them to unburden and feel “lighter”. 

 Many of the participants mentioned that members in their respective groups had reached out to each other and broken social barriers in doing so and shared some of these experiences in the FGD. For example, one of the participants said that in her group, a member had shared her story about her husband's chronic illness and his subsequent inability to work. She lived with her mother-in-law, husband, and two children and found it difficult now to make both ends meet just from the income from the land. On hearing about her dire economic circumstances, the group members suggested that she could start a small business to supplement the family agricultural income. To this, the member responded that her spouse and mother-in-law were against the idea as women of her community did not engage in economic activity outside of the house. The group members encouraged her to start a small business of selling food products that she could make in the house and helped her to market it. When she began to earn money, the family supported her decision to expand the business and hire another person to market the products. Another participant mentioned that in her group, a member was concerned about the future of her daughter who had been widowed at a young age. The members heard her out and then supported her decision to find a groom for her daughter, much against the wishes of the elders in the family. 

Participants were asked whether the nature of the interaction among members had changed in the SHG after the introduction of the mental health intervention. They generally indicated that prior to the introduction of the mental health intervention, they rarely discussed personal or family concerns with each other. The SHG meeting would be conducted in a matter-of-fact way and wound up in about half an hour so as to not waste anyone's time. The interaction would be more competitive with each member making a case that her need for a loan was greater than that of the others. However, now that they knew each other's personal life situation and understood each other better, they found it easier to prioritize whose need was more and disburse the loan amount equitably and amicably in a cooperative manner. The women also stated that they now took keen interest in each other's economic activities and gave each other suggestions to improve the same. This was supported by the fact that in Group I (with the mental health intervention), 85% of the women had started a small business or additional income generating activity, while in Group II (without the mental health intervention) only 40% of the women had done so. The women added that although the mental health meeting took at least an hour, no one felt it was a waste of time, since they valued the inputs from the members.

Participants' unanimously stated that there was a high level of trust and a kinship that they now shared with their group members. The support shown by group members for each other made them feel that they were not alone and could face any life situation. The experience of the senior members of the group, combined with the wisdom of the younger ones who were able to quickly learn new things, generated a good mix of coping strategies. Group members no longer worried or felt distressed over a problematic situation for too long, because they knew they could share it in the group and be reassured and supported or helped to find an appropriate solution.

## 5. Discussion

The experience of combining income generation through microcredit activity and reduction of psychological distress through a group-based mental health intervention has been both challenging and rewarding. The process of capacity building using the SHG model had strengthened and enhanced rural women's ability to generate income. This had led to increased economic security, since they now had a safety net to fall back on in times of need. Several studies have documented the efficacy of the SHG model as a poverty alleviation strategy [2, 16]. Contributing to the family income also resulted in these women commanding more attention and respect within the household and being involved in decision making processes, both within and outside of the family. This finding is borne out by other studies carried out with women in the microcredit sector [20]. 

However, the addition of the mental health intervention to the SHG microcredit activity while addressing the psychological concerns of members and reducing their distress, also resulted in greater interpersonal trust, a more cooperative spirit and a stronger social support network. Interpersonal trust and reciprocity within social networks is said to be key to the strengthening of social capital which, in turn, is linked to enhanced well-being [21]. 

Other studies have indicated that while SHG participation may improve access to health care, it may not be sufficient to address the mental health concerns of poor women [2, 22]. The present study demonstrates that at an initial cost of about $13 per woman, it was possible to put a mental health intervention in place that addressed women's emotional needs, improved their access to health related information and health services, strengthened their interpersonal bonds, and enhanced their well-being. Several researchers have commented that development interventions need to be integrative so that they address poverty alleviation, strengthen social capital, and empower the poor [23–25]. 

There were several challenges faced in implementing the mental health intervention. Finding a clean, quiet, and private space that women could easily access proved to be a major challenge. SHG meetings for economic activity alone often took place in a member's house with all the members sitting huddled together. A larger and more private space for the mental health meetings was required to practice the relaxation technique as well as discuss personal and family matters. Public spaces such as school corridors, temple courtyards, or government office buildings with a porch/portico were identified for this purpose. Women usually conducted these meetings at night and often had to reschedule the meeting if there was a power failure.

The present study is an attempt to share a novel and challenging experience of working with extremely underprivileged rural women and has several limitations. The small sample size does not permit any generalizations. The lack of literacy skills ruled out the possibility of using any kind of quantitative assessment such as rating scales. The qualitative data obtained by the use of FGDs was also not very rich in terms of thematic content, due to the limited capacity for verbal expression in the participants. Members did not openly disagree or express opinions of dissent and often readily agreed with the first opinion that was voiced by a participant. They had to be actively encouraged to express their individual opinions and often responses to open-ended questions were reduced to a show of hands. However, voluntary participation, regular attendance in the sessions (an average attendance rate of about 70%), and positive feedback from the members indicate user satisfaction with the mental health intervention. Leaders of SHGs that had participated in the mental health intervention shared their experiences with other SHG leaders in their monthly cluster meetings. This has resulted in the leaders of the other SHGs requesting Sampark to introduce the intervention for their groups as well. Finally, and most importantly, the women have taken ownership of the program and are ensuring its sustainability by continuing to meet on a fortnightly basis for the mental health intervention. These are qualitative indicators that adding the mental health intervention to the ongoing economic activity in the SHG has made a positive difference in these women's lives.

## 6. Conclusions

Microcredit and microenterprise activities have been instrumental in reaching out to very poor and marginalized persons, especially women, in developing countries. This has led to increase in income generating capacity and economic security. However, health, including mental health, influences participation in economic activity and earnings. Mental health interventions in the context of poor, underserved regions, such as rural India, are particularly challenging. Community-based mental health interventions serve not only to reduce psychological distress, but also strengthen women's social capital. Building strong interpersonal networks is an important psychological resource that enhances emotional well-being. SHG participation can be used as a platform to provide women with access to health care and promote their mental health. The present study provided qualitative evidence that the mental health intervention had been well received by the women. Therefore, Sampark intends to extend the mental health intervention to all its SHGs and make it an integral component of its poverty alleviation programme.

## Appendices

### A. Training of Counselors

The initial module, for the training of Sampark staff as lay counselors was of one week duration. The following topics were covered in the training:

- basic introduction to the human body and its physiology,
- understanding the relationship of body and mind,
- recognizing common physical ailments and symptoms,
- identifying mental disorders,
- lifestyle management: personal care and hygiene,
- lifestyle management: diet and activity,
- introduction to counseling and communication skills,
- techniques of counseling including active listening, open/closed questions, ventilation, acceptance, reassurance, guidance, reflection,
- ethics of counseling and qualities of a counselor,
- coping skills: an introduction,
- coping skills: problem solving skills (cognitive appraisal, restructuring, problem-solving),
- coping skills: emotion focused skills (denial, distraction, religion, humor, acceptance).

The pedagogy of training included didactic presentations and participatory exercises such as role playing, story telling, and group activities. Participants were given homework assignment and handouts and other reading material for further study.

A breathing-based relaxation exercise using diaphragmatic breathing was demonstrated and then practiced twice a day, at the end of the morning and afternoon session, to ensure that the counselors were proficient in the practice.

The counselors then conducted four pilot counseling sessions in the SHGs with the trainers as observers. They maintained detailed notes of their sessions and noted the difficulties they experienced during these sessions. A second training program of two-day duration was conducted during which the mental health professionals discussed the experiences of the counselors, gave feedback, and provided further inputs to improve their skills.

The counselors maintain notes of each group session that they conduct. They are provided periodic supervision and booster training by a mental health professional.

The lay counselors, in turn, with supervisory support from the mental health professionals, conduct the training for the group facilitators identified by the SHG members. This training is imparted over 2 two-day workshops using more practical exercises and role plays and less of didactic pedagogy.

### B. Mental Health Intervention

Session 1 The objectives, purpose, and ethical considerations of the mental health intervention are introduced in the first session. General health and personal hygiene related issues are discussed.

Session 2 The session provides psychoeducation on mind and body relationships and how psychological problems and illness affect the body and vice-versa.

Session 3–8 These six sessions are the main counseling sessions where the group counselor facilitates the sharing of problems, reviews coping strategies, and encourages alternate and more adaptive ways of problem solving and coping. The focus is to encourage the group members to ventilate their problems and support and help each other in the process.

Session 9 and 10 The group counselor prepares the group in Session 9 for the termination of sessions by the counselor after Session 10. The counselor encourages and facilitates the group to continue to meet on a fortnightly basis. The group identifies two facilitators from amongst themselves, who then undergo training in basic counseling and facilitation skills.

Session 11 and 12 The counselor observes the 11th session carried out by the facilitator chosen by the group. The 12th session is conducted by the counselor as a booster session and observed by the facilitator. Subsequently, the group continues to meet on its own and the facilitators report to the counselors for supervision and further training inputs.
